# Effects of rock wool on the lungs evaluated by magnetometry and biopersistence test

**DOI:** 10.1186/1745-6673-4-5

**Published:** 2009-03-27

**Authors:** Yuichiro Kudo, Makoto Kotani, Masayuki Tomita, Yoshiharu Aizawa

**Affiliations:** 1 Department of Preventive Medicine and Public Health, Kitasato University School of Medicine, 1-15-1, Kitasato, Sagamihara, Kanagawa 228-8555, Japan; 2 NICHIAS Corporation, 1-26, Shibadaimon 1-chome, Minato-ku, Tokyo 105-8555, Japan

## Abstract

**Background:**

Asbestos has been reported to cause pulmonary fibrosis, and its use has been banned all over the world. The related industries are facing an urgent need to develop a safer fibrous substance. Rock wool (RW), a kind of asbestos substitute, is widely used in the construction industry. In order to evaluate the safety of RW, we performed a nose-only inhalation exposure study in rats. After one-month observation period, the potential of RW fibers to cause pulmonary toxicity was evaluated based on lung magnetometry findings, pulmonary biopersistence, and pneumopathology.

**Methods:**

Using the nose-only inhalation exposure system, 6 male Fischer 344 rats (6 to 10 weeks old) were exposed to RW fibers at a target fiber concentration of 100 fibers/cm^3 ^(length [L] > 20 μm) for 6 hours daily, for 5 consecutive days. As a magnetometric indicator, 3 mg of triiron tetraoxide suspended in 0.2 mL of physiological saline was intratracheally administered after RW exposure to these rats and 6 unexposed rats (controls). During one second magnetization in 50 mT external magnetic field, all magnetic particles were aligned, and immediately afterwards the strength of their remanent magnetic field in the rat lungs was measured in both groups. Magnetization and measurement of the decay (relaxation) of this remanent magnetic field was performed over 40 minutes on 1, 3, 14, and 28 days after RW exposure, and reflected cytoskeleton dependent intracellular transport within macrophages in the lung. Similarly, 24 and 12 male Fisher 344-rats were used for biopersistence test and pathologic evaluation, respectively.

**Results:**

In the lung magnetometric evaluation, biopersistence test and pathological evaluation, the arithmetic mean value of the total fiber concentration was 650.2, 344.7 and 390.7 fibers/cm^3^, respectively, and 156.6, 93.1 and 95.0 fibers/cm^3 ^for fibers with L > 20 μm, respectively. The lung magnetometric evaluation revealed that impaired relaxation indicating cytoskeletal toxicity did not occur in the RW exposure group. In addition, clearance of the magnetic tracer particles was not significantly affected by the RW exposure. No effects on lung pathology were noted after RW exposure.

**Conclusion:**

These findings indicate that RW exposure is unlikely to cause pulmonary toxicity within four weeks period. Lung magnetometry studies involving long-term exposure and observation will be necessary to ensure the safety of RW.

## Background

Rock wool (RW) is a kind of asbestos substitute and is widely used in the construction industry, in particular for fire-resisting insulation, thermal insulation, and acoustic absorption. However, some asbestos substitutes, including RW fibers, resemble asbestos morphologically, and their possible harmful effects on humans have been a concern. Pulmonary fibrosis has occurred in rats experimentally exposed to RW, but no development of lung tumors was noted [1]. Regarding the safety of RW, the International Agency for Research on Cancer (IARC) at present classifies RW as Group 3: limited evidence in experimental animals for the carcinogenicity, and inadequate evidence in humans for the carcinogenicity [2,3].

Lung magnetometry was first performed by Cohen in 1973 [4]. The primary feature of this method is that this is an in vivo test of the living organism, and the proper function of the main defense cell in the lung (macrophages) can be non-invasively monitored. Using this method, we can obtain knowledge about the intracellular movement of alveolar macrophages, after making them to ingest magnetic particles, by measuring the remanent magnetic field strength in the lung after external magnetization. Since the ingested magnetic particles remain in the phagosomes, intracellular movement of the phagosomes can be detected by measurement of remnant magnetic field [5-7].

To date, we have evaluated the cytotoxicity of chrysotile, a type of asbestos, as well as RW and other man-made vitreous fibers (MMVFs), by cell magnetometry that was originally devised in our laboratory [8-12]. This method determines cytoskeleton-dependent functions of macrophages, which play an important role in phagocytosis, to evaluate the degree of injury caused on macrophages. In our previous report, the cell magnetometric evaluation revealed that RW is less cytotoxic than chrysotiles [11].

Biological effects of MMVFs need to be evaluated not only at the cell level but also in the lung. To our knowledge, however, there have been no studies to evaluate the safety of RW by means of lung magnetometry. We thus performed the present study with the aim of evaluating the potential of RW to cause pulmonary injury. In this study, rats were forced to inhale RW by a nose-only inhalation exposure system, then evaluated by lung magnetometry, biopersistence test (changes over time in the number and size of fibers that retained in the lungs) and pathological examination.

## Methods

The present study was performed in accordance with the Ethical Guidelines for Animal Experimentation adopted by the Institutional Review Board of Kitasato University School of Medicine (Approval No. 2004022).

### Materials

As an experimental material, we used an RW sample manufactured by NC Co., Ltd., Japan that was provided by the Rock Wool Association, Japan. Fluorescence X-ray spectroscopy showed that the RW used in the present study was chemically composed of SiO_2 _39%, CaO 33%, Al_2_O_3 _14%, MgO 5.0%, Fe_2_O_3 _1.8%, and S 0.6%.

Originally, RW is present in the form of lumps of different fiber sizes (both length and width). We adjusted the RW fiber size in accordance with the method of Kohyama et al. (1997) to obtain fiber samples of appropriate size for animal experiments [13]. RW fibers thus obtained were dispersed in an exposure chamber and the fiber sizes were measured. Their geometric mean length (geometric standard deviation, GSD) and geometric mean width (GSD) were 15.49 (2.02) μm and 2.44 (1.59) μm, respectively. Then, to make it easier to generate RW in the nose-only inhalation exposure system, the pressurized and pulverized RW fibers were mixed with glass beads (BZ-02, AS ONE Corporation, Osaka, Japan) at a weight ratio of 1 (RW) to 39 (glass beads).

### Exposure study

Male Fischer 344 (F344) rats (6 to 10 weeks old; which is specifically recommended by EC Protocol, 1999) were used for each experiment. To acclimatize the rats to the environment of the laboratory, they were first housed in cages for about one week with free access to water and food. The temperature was kept at 22°C and 40% humidity, with a continuous supply of fresh filtered air.

In the lung magnetometric evaluation, an exposure group and a control group comprised 6 rats each. The study material (RW fibers) was supplied with air into the exposure chamber and exposed to the noses of rats of the exposure group in the same way as reported previously [14-16]. The rats in the control group were not exposed to RW but underwent lung magnetometry only.

In the biopersistence test, 12 rats were used per experiment and the experimental was repeated twice, and in phathological evaluation, 12 rats were used (36 rats in total). The rats were exposed to RW fibers continuously for 6 hours daily for 5 consecutive days. Each day during the experimental period, the rats fixed in the upper rat holders of the main chamber were replaced by the rats in the lower rat holders, rotating the positions among the upper and lower rat holders.

### Lung magnetometry

**Figure 1:**
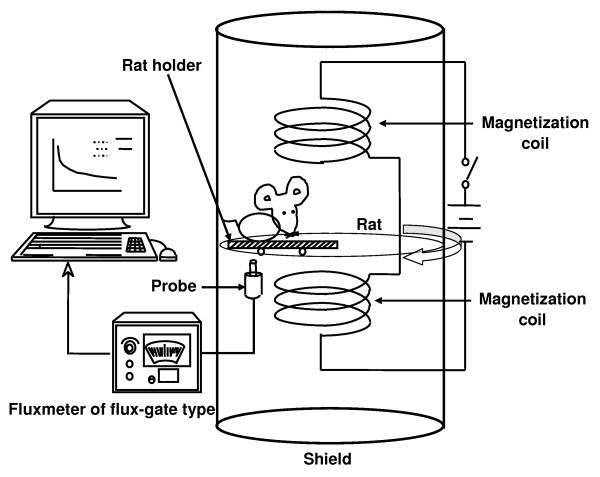
**Lung magnetometric evaluation apparatus**.

Figure 1 shows an outlined view of the lung magnetometric evaluation apparatus. Magnetometric evaluation of lungs was performed in 6 rats each of RW-exposed and control groups according to the method reported by Aizawa et al. (1991). One day after exposure, rats were anesthetized by inhalation of diethyl ether. In this study triiron tetraoxide (Toda Kogyo Corp., Tokyo, Japan) was used as magnetic particles with the geometric mean particle size of 0.26 μm.

RW-exposed and control rats were intratracheally catheterized and instillated with 3 mg of triiron tetraoxide suspended in 0.2 mL of physiological saline one day after RW exposure. Each rat was then anesthetized with intraperitoneal Nembutal (at 0.15 mL/100 g body weight). Magnetization for one second was performed to the rat chest under a magnetic flux density of 50 mT, followed by a 40-minute measurement of strength of the postmagnetization remanent magnetic field with a fluxmeter of flux-gate type. The apparatus was operated in such a way that the sample table passed over the probe once every 12 seconds. Magnetization and measurement of the remanent magnetic field of the lung was performed 1, 3, 14, and 28 days after RW exposure. By measuring the remanent magnetic field over 40 minutes postmagnetization, a curve indicating the decay constant can be obtained. Further, measurement of the remnant magnetic field strength for 2 min postmagnetization gave a nearly linear curve when plotted after logarithmic transformation. The point at which this curve intersected with the y-axis was designated B_0_. When expressing the remnant magnetic field immediately after magnetization as B_0 _and the decay constant as λ, the remnant magnetic field after t seconds of termination of external magnetization can be represented by the formula B = B_0_e^-λt^, and thus the decay constant (λ) was calculated based on this formula. In addition, the maximum strength of remanent magnetic field on each measurement day (t = 0 - minute value) was calculated with the value on Day 0 taken as 100%, on the basis of which clearance curves were prepared.

### Biopersistence test

One, 3, 14 and 28 days after exposure, 6 rats were sacrificed a time (1D group, 3D group, 14D group, and 28D group, respectively). Rats were weighed once every week. During and after exposure, rats were intermittently monitored for any change in their appearance or condition.

Under Nembutal anesthesia, rats were sacrificed by exsanguination from the abdominal aorta and their lungs were resected. The resected lungs were ashed in a low-temperature asher (Plasma Asher, LTA-102, Yanaco Corp., Kyoto, Japan) over 24 hours.

The ashed specimen containing fibers was suspended in distilled water that had been filtered with a Minisart (Sartotius K. K., Tokyo, Japan) syringe filter unit in a weighing bottle. Fibers were collected on a Nuclepore filter (pore diameter, 0.2 μm) using a suction filter, and allowed to dry. At least 400 fibers were counted for each rat by use of a scanning electron microscope (BX41, Olympus Corp., Tokyo, Japan) at ×500 to ×2000 magnification. Fibers counted were those having an aspect ratio (ratio of length to width) of 3 or greater. The number of fibers in each of the three categories of length (L) (L ≤ 5, 5 < L ≤ 20, or L > 20) was obtained in accordance with the rules for fiber counting [17]. Among the fibers counted, World Health Organization (WHO) fibers – which have a length of longer than 5 μm and a width of shorter than 3 μm [2] – were also counted. The fiber number was then converted to the fiber number per weight of dried lung. The half-life of fibers in the rat lungs was calculated assuming that the geometric mean of the total fiber number/the total lung weight (fibers/mg) in the lungs of the 1D group was 100% [3].

Furthermore, the fiber size (length and width) was measured at ×500 to ×2000 magnification. In this measurement, fibers having a length of 0.47 μm or greater and a width of 0.05 μm or greater were included.

### Pathological evaluation

Three rats each were sacrificed 1, 3, 14, and 28 days after RW exposure. Their lungs were isolated and fixed in formalin, followed by observation of lung tissue by hematoxylin and eosin staining using a transmission electron microscope.

### Statistical analysis

In the lung magnetometric evaluation, arithmetic mean values and standard deviations were calculated from data obtained for the RW-exposed and control groups of 6 rats each. Subsequently, Students' t-test was conducted.

In the biopersistence test, geometric mean and geometric standard deviation were calculated for the total fiber number, length and width. For length and width, at least 400 fibers in lungs per rat were counted in two experiments and the geometric mean value for 6 rats was calculated. One-way analysis of variance was performed and Scheffe's multiple comparison test was conducted.

## Results

### Fiber concentration and weight concentration in exposure chamber

In the lung magnetometric evaluation, the arithmetic mean (standard deviation, SD) of the total fiber concentration in the exposure chamber during the experiment was 650.2 (367.3) fibers/cm^3^, and 156.6 (104.7) fibers/cm^3 ^for fibers with L > 20 μm. The arithmetic mean (SD) of the weight concentration was 170.4 (29.3) mg/m^3^.

In the biopersistence test, the arithmetic mean (SD) of the fiber concentration was 344.7 (161.6) fibers/cm^3 ^for all fibers and 93.1 (50.2) fibers/cm^3 ^for fibers with L > 20 μm. The arithmetic mean (SD) of the weight concentration was 100.0 (29.9) mg/m^3^.

In the pathological evaluation, the arithmetic mean (SD) of the fiber concentration was 390.7 (170.4) fibers/cm^3 ^for all fibers and 95.0 (45.8) fibers/cm^3 ^for fibers with L > 20 μm. The arithmetic mean (SD) of the weight concentration was 100.2 (26.4) mg/m^3^.

### Lung magnetometry

**Figure 2:**
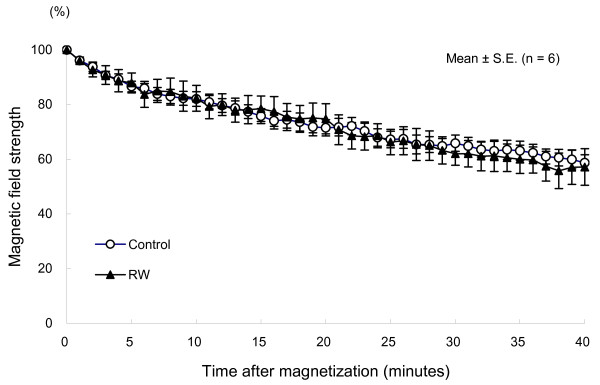
**Relaxation of triiron tetraoxide microparticles in the lung**. In both the RW-exposed and control groups, relaxation was rapid on all measurement days.

**Figure 3:**
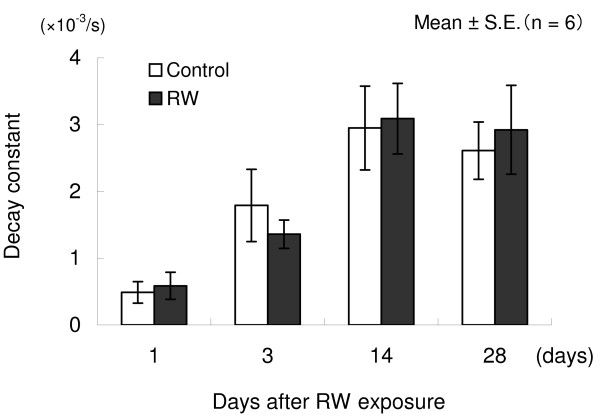
**Changes over time in decay constant after infiltration of triiron tetraoxide particles**. The decay constant during a 2-minute postmagnetization period did not significantly differ between the two groups on any measurement day.

Assuming that the percentage of remanent magnetic field strength immediately after magnetization on each measurement day was 100%, the percentages of 40-minute postmagnetization period were calculated and plotted to construct relaxation curves. In both the RW-exposed and control groups, relaxation was rapid on all measurement days, as shown in Figure 2. No significant differences in decay were noted between the two groups on any of the study days. Between day 1 and day 14 there was an increase in the decay constant indicating an acceleration of relaxation during this time period. The decay constant during a 2-minute postmagnetization period did not significantly differ between the two groups on any measurement day (Figure 3).

**Figure 4:**
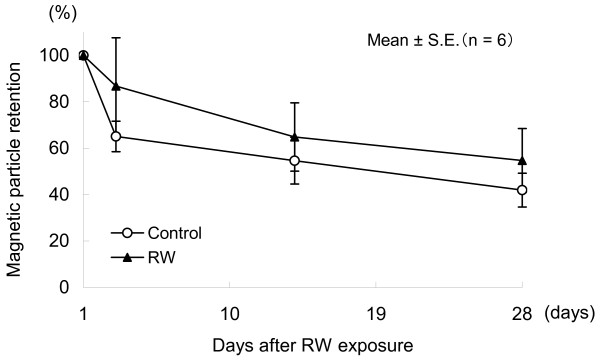
**Clearance of iron particles from rat lungs determined by lung magnetometry**. Both the RW-exposed and control groups showed rapid magnetic particle clearance.

The percentage of the remanent magnetic field strength immediately after magnetization (B_0_) on each measurement day was calculated with the value obtained one day after exposure taken as 100%. The decay of B_0 _shows the retention and clearance of the magnetic particles in the lung. Both the RW-exposed and control groups showed rapid magnetic particle clearance. In the RW-exposed group, however, magnetic particle clearance was impaired in tendency (Figure 4).

### Biopersistence test

**Figure 5:**
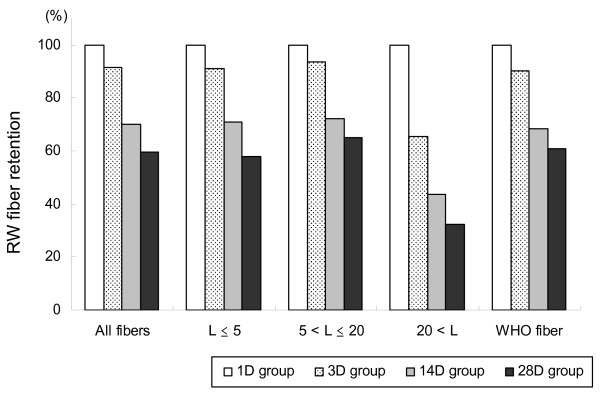
**Changes in the intrapulmonary fiber count over time**. The percentage of the number of fibers retained in the lungs in each group calculated with the geometric mean of the 1D group taken as 100%.

Table 1 shows the changes over time in the number of RW fibers that retained in lungs, and Figure 5 shows the percentage of the number of the retained fibers, calculated with the geometric mean of the 1D group taken as 100%. The total fiber number, fiber number by size, and WHO fiber number decreased over time in the observation period. The results of Scheffe's multiple comparisons showed that the fiber number in all categories significantly decreased in the 28D group as compared with the 1D group (p < 0.05) (Table 1).

**Table 1:** Geometric mean of number of fibers retained in the lung (geometric standard deviation)

Observation period	All fibers	L ≤ 5	5 < L ≤ 20	20 < L	WHO fibers
	10E5 fibers/g dry lung weight				

1D group	93.3 (1.2)	45.6 (1.2)	41.4 (1.2)	6.2 (1.3)	47.6 (1.2)
3D group	85.6 (1.2)	41.5 (1.2)	38.8 (1.3)	4.1 (1.7)	43.0 (1.4)
14D group	65.5 (1.1)^a^	32.2 (1.2)^a^	29.8 (1.2)	2.7 (1.7)^a^	32.7 (1.3)
28D group	55.6 (1.2)^a, *b*^	26.4 (1.3)^a, *b*^	26.9 (1.1)^a, *b*^	2.0 (1.3)^a^	29.0 (1.1)^a, *b*^

^a^: Comparison with the 1D group (p < 0.05)

^b^: Comparison with the 3D group (p < 0.05)

L = Fiber length (μm)

WHO fibers: Fibers having a length of longer than 5 μm and width of shorter than 3 μm.

The half-lives of RW fibers were calculated from an exponential approximation curve after the geometric mean of the 1D group was taken as 100%. The half-lives were 35 days for all fibers, 16 days for the fibers with L > 20 μm, and 35 days for WHO fibers. These findings indicate that the half-life of RW fibers with L > 20 μm was shorter (16 days) than that of all fibers or WHO fibers (35 days), showing that RW fibers have lower biopersistence.

As shown in Table 2, both length and width reduced over time in the observation period. Upon Scheffe's multiple comparisons, the 3D and 28D groups showed significantly shorter widths than the 1D group (p < 0.05) (Table 2).

**Table 2:** Changes in length and width of fibers over time

Observation period	Length	Width
	
	Geometric mean (Geometric standard deviation) (μm)	
1D group	5.52 (2.48)	0.50 (1.86)
3D group	5.28 (2.38)*	0.48 (1.90)*
14D group	5.29 (2.30)	0.48 (1.91)
28D group	5.29 (2.22)*	0.48 (1.97)*

*: Comparison with the 1D group (p < 0.05)

### Pathological evaluation

An electron microscope image of the lung in the 28D group showed that macrophages retained morphologically almost normal nuclei and cytoplasm. Lung tissues did not show pulmonary fibrosis and were almost normal (data not shown).

## Discussion

The principle of lung magnetometry is to apply external magnetization to lungs in which magnetic particles are retained. After withdrawal of the external magnetization, a weak remanent magnetic field of the lung can be detected. Rapid decay of the remanent magnetic field following withdrawal of magnetization is called relaxation. Triiron tetraoxide phagocytosed by alveolar macrophages is magnetized by external magnetization and arranged so as to be orderly aligned in a single direction, and after withdrawal of external magnetization, the phagosomes rotate cytoskeleton-dependently at random, resulting in rapid decay of the remanent magnetic field. When a toxic substance capable of causing pulmonary injury is administered, however, the substance may have physical and/or chemical effects on the cytoskeleton, impairing phagosome motion and retarded decay of the magnetic lung field. This slower rotation means that it is less likely for magnetic particles to deviate from the alignment, which may result in delayed relaxation.

Relaxation is only noted in living bodies and not observed in autopsied lungs or lungs isolated from dead animals. Accordingly, lung magnetometry enables noninvasive evaluation of pulmonary toxicity in living subjects. In addition, measurement of remanent magnetic field strength from immediately after external magnetization to a subsequent follow-up period allows estimation of time-course changes (clearance) in the quantity of persistent magnetic particles in lungs. When a lung-toxic substance is simultaneously administered, the clearance of magnetic particles is delayed, which enables the determination of whether or not the substance is responsible for lung injury. The decay constant indicates the degree of cytotoxicity: the greater this value, the smaller the cytotoxicity.

Aizawa et al. performed studies using lung magnetometry, in which gallium arsenide or silica was intratracheally administered in rabbits, indicating that relaxation and clearance were delayed in a dose-dependent manner [18,19].

The relaxation curves obtained in the present study did not significantly differ between the RW-exposed and control groups, showing rapid relaxation in both groups. It is considered that after RW exposure, phagosomes could be efficiently transported along the cytoskelton, resulting in rapid relaxation.

The decay constant was determined for two minutes following withdrawal of magnetization, during which the remanent magnetic field usually rapidly reduces. The greater the value, the more rapid is the relaxation. Between the RW-exposed and control groups, no significant difference was observed in decay constant, which may indicate that phagosomes rapidly rotated even after RW exposure, as shown by the relaxation curves. The decay constant increased with time, reflecting faster relaxation and uptake of magnetic particles by macrophages. There may be another possibility of decrease in magnetic particle size (smaller particles show faster relaxation).

The clearance curves revealed that the remanent magnetic field strength – indicating the quantity of magnetic particles retained in lungs – determined immediately after magnetization in the RW-exposed group reduced over time as well as in the control group, showing no significant differences between the two groups. These findings may indicate that exposure to RW did not influence the defense and clearance mechanisms in the lung.

In the biopersistence test, the number and sizes (length and width) of fibers persisting in lungs decreased from one day to 28 days after exposure. The reduction in the number of persisting fibers may be related to excretion of fibers by mucociliary movement or phagocytosis of fibers by alveolar macrophages, and the reduced sizes (length and width) of fibers retained in the lungs may be due to dissolution in body fluid or mechanical destruction of fibers [3]. The reason why longer fibers clear faster compared to shorter fibers is considered to be that longer fibers may primarily deposit in the airways and follow mucociliary clearance while shorter fibers penetrate deeper into the lung periphery. Pathological evaluation revealed no obvious changes.

## Conclusion

The findings of the present study suggest that RW exposure may not cause significant lung toxicity within four weeks period. To further ensure the safety of RW, lung toxicity should be evaluated for at least one year after RW exposure, in which we are engaged in our ongoing study.

## Competing interests

The authors declare that they have no competing interests.

## Authors' contributions

YK and MT have made substantial contributions to conception and design, acquisition of data, and analysis and interpretation of the data. MK has been involved in drafting the manuscript and revising it critically for important intellectual content. YA have given final approval of the version to be published. All authors read and approved the final manuscript.
